# Decolonizing global health: what should be the target of this movement and where does it lead us?

**DOI:** 10.1186/s41256-022-00237-3

**Published:** 2022-01-24

**Authors:** Xiaoxiao Kwete, Kun Tang, Lucy Chen, Ran Ren, Qi Chen, Zhenru Wu, Yi Cai, Hao Li

**Affiliations:** 1 Global Health Research and Consulting, Yaozhi, Yangzhou, China; 2 Vanke School of Public Health, Tsinghua University, Beijing, China; 3 National School of Development, Peking University, Beijing, China; 4 Global Health Research Center, Dalian Medical University, Dalian, China; 5 Institute for Advanced Studies in Humanities and Social Sciences, Beihang University, Beijing, China; 6 Institute for the World Economy, Shanghai Academy of Social Sciences, Shanghai, China; 7 Global Health Institute, Wuhan University, Wuhan, China

**Keywords:** Decolonization, Global health, Post-colonialism

## Abstract

The current decolonizing global health movement is calling us to take a post-colonial perspective at the research and practice of global health, an area that has been re-defined by contemporary scholars and advocates with the purpose of promoting equity and justice. In this article, we summarize the main points of discussion from the Symposium organized by the editorial board of Global Health Research and Policy, convened in July 2021 in Wuhan, China. Experts participating in the symposium discussed what decolonizing global health means, how to decolonize it, and what criteria to apply in measuring its completion. Through the meeting, a consensus was reached that the current status quo of global health is still replete with various forms of colonial vestiges–ideologies and practices–, and to fully decolonize global health, systemic reforms must be taken that target the fundamental assumptions of global health: does investment in global health bring socioeconomic development, or is it the other way around? Three levels of colonial vestiges in global health were raised and one guiding principle was proposed when thinking of solutions for them. More theoretical discussion needs to be explored to guide practices to decolonize global health.

## Background

The term “global health” was defined by various scholars, such as Kickbush (2006), Macfarlane (2008), Koplan (2009), Chen et al. (2020), etc., at the start of this century, as an area that focused on health issues with a global concern and with the goal of promoting global health equity [1–5]. However, the area of practice and research that constitutes what’s now called global health has long existed since the colonial era and is deeply rooted in the necessity to understand the disease etiology, pattern, and treatment of the indigenous populations living in the Southern hemisphere, which was then given the name “tropical medicine” [2, 6]. At that time, the objectives of both practice and research were built to protect the interests of colonizers, usually against the interests of the colonized [7, 8]. The sprouts of emancipatory movements in the mid-twentieth century brought a large number of countries in Asia, Latin America and especially Africa to their independence within a relatively short period [9]. Colonizers were pushed out of their colonized territories and bilateral aid agencies were born around the same time, many, from former agencies that managed colonial assets for their regime [10, 11]. The successful role the Marshall Plan played in the reconstruction of Europe [12] gave the world a promising future where aid money produced effective and sustainable development, a future we have yet to see in the global south. Nonetheless, large sums of Official Development Assistance (ODA) have been flowing from those former colonizers to former colonized countries for the latter’s development, which were later also used by the United States of America and the Soviet Union to attract countries to join their respective blocs during the Cold War [13]. During this time, those practices and research were called “international health”, a term that focuses on the cooperation and exchange of resource and power among sovereign nations based on their respective needs. However, the funds didn’t come without costs for the recipient countries. From the 1970s, The World Bank (WB) and International Monetary Fund (IMF) started an approach known as Structural Adjustment: countries who want to borrow money from them during crises have to follow very strict austerity as stipulated by the WB and IMF as necessary for the economic recovery [14]. Those measures include cutting public sector budgets, such as salaries for doctors and teachers. On the other hand, top young talents in the health sector are quickly brain-drained out of low- and middle- income countries (LMICs) as many high-income countries have preferrable policies for attracting those talents to work or even immigrate [15]. As a result, the health system of recipient countries is weakened, further limiting the space for growth [16]. International health thus served as a sweetener in a package deal that perpetrated the unjust system facing former colonized countries.

In the twenty-first century, global health has been framed as a call for equity and justice [2]. However, the colonial remnant are apparent such that it could be argued that more often than not, global health is old wine in a new bottle. In this paper we offer evidence and reasons for holding this position. We need to recognize that up until today, we are observing many “legacies” from the colonial times, and the ongoing decolonizing global health movement is a reminder that only by completely removing those “legacies” can global health truly embrace its new life in the spirit of global solidarity. Building on years of critical reflection within the global health community from a post-colonial perspective [6, 8], this movement gained momentum from a few key publication in 2018 [8, 17, 18], and was amplified by student conferences across major universities in the developed world [19–22], with continuous social media engagements from beyond academia [23]. By March 2021, major global health journals have joined the discussion, publishing articles ranging from a general description of what a decolonized global health would look like [24, 25] to focused discussion on a specific topic such as the choice of terms [26]. All seem to recognize that the status quo in global health does not offer fair opportunities for LMICs, and to identify the root cause, one must go back to the colonial era when resources were taken out of Africa, Asia, and Latin America by coercion or outright brute force.

**Fig. 1:**
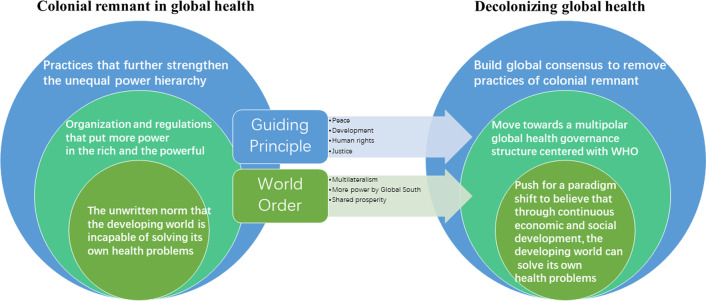
Three levels of colonial remnant in global health and their corresponding solutions

Decolonization as a process is still an unfinished agenda in today’s world and global health is a mere reflection of this fact. While many scholars have explored various facets of today’s world from a post-colonial and neo-colonial perspective [27], most of their discussions have been limited to the area of literature and political sciences. Few have taken a systemic theoretical approach to the post-colonial discussion in global health. In this article, we attempt to take a step toward a systemic approach and categorize the colonial remnant in global health today that this decolonization movement should target into three levels: at the level of practices, at the institutional/organizational level and at the policy level for a fundamental paradigm shift (Fig. 1).

## Three levels of colonial remnant in global health and their corresponding solutions

### In daily practices

The colonial remnant in the daily practice of global health has been well-recorded in the recent discussions of decolonizing global health. In his publication on The Lancet, Dr. Seye Abimbola described a world where global health has been decolonized. In this world, authors from LMICs have the same publication opportunities as those from high-income countries (HICs), and technical support is also sent by LMICs to high income countries (HICs) in terms of financial resources and expert teams [24]. However, what we observe in today’s global health indicates that this description is no better than wishful thinking: many aid for health projects are still donor driven and not locally owned, despite strong advocate for the contrary from global health scholars and recipient governments. HICs are also home to the vast majority of the world’s best institutes for training, education and research in global health, the world’s top journals on global health, and the world’s most impactful International Civil Society Organizations (ICSOs) and private foundations. Almost all current campaigns in global health and related fields, such as the climate change, universal health coverage, and the previous campaigns on human immunodeficiency virus infection and acquired immune deficiency syndrome (HIV/AIDs), tuberculosis (TB) and malaria are all initiated and led by people born and raised in HICs. It is promising to see that, China, recovered from successful decolonization in history, has showed its leadership in global solidarity and fights against the COVID-19 pandemic [28].

After the Paris Declaration on Aid Effectiveness, the Accra Agenda for Action and the Busan conference [29, 30], global health society has set up different mechanisms to push for external funding for health to be better aligned with local priorities. One of them is to call for all donors to invest in areas set forth in the national Health Strategic Development Plans (HSDPs), which are published by local countries to guide all foreign and domestic investments to avoid overlaps and neglected areas. However, then, in many extreme resource constraint countries, the national HSDPs have been drafted and led by international organizations such as the World Bank. Donor interested projects would get written in this document much faster than those proposed by local governments [31]. As a result, global health society has been mainly focusing on controlling infectious diseases such HIV/AIDs, malaria, TB and others. Fundings for non-communicable diseases (NCDs) such as hypertension and diabetes remain very limited, despite the fact that the majority of the disease burden for LMICs, including Latin America and South East Asia, are from NCDs [32].

The list of global health practices that are colonial legacies is long; it includes anything that perpetuates the inferior status of the people and system on the receiving ends of the global health services. It comes from sponsors who give grants and build capacities to increase channels for imposing donor control, researchers who conduct research to instigate racial or ethnic disputes at the expense of the population they study [33], and practitioners who deliver healthcare in a way that further cripples the local health care system and make the people more dependent on external help. Unfortunately, those practices are rather prevalent in global health today with experts and scholars being sent to areas of extreme resource scarcity where no locals can sustain the projects once they leave. New global consensus needs to be reached on what constitutes a success in global health to promote true sustainable changes of local communities.

### Organizational and regulatory inertia/obstacles

Shortly after it became clear that the COVID-19 outbreak had hit the economy hard last year, the UK government cut the budget of the Department For International Development by about 4 billion dollars a year; many global health projects suffered tremendously [34]. During Trump’s 4-year administration, multiple proposals were made from the President’s Office to cut the budget for foreign aid and global health because they were incompatible with his foreign policy for the American people [35]. Both show a fundamental mismatch in the global health world: aid agencies and private foundations serve people in the developing world, but they are ultimately held accountable for and by the people in the developed world. The setup in itself resembles that in the colonial times.

After the Second World War, multilateral institutions were set up to prevent the world from going into a Third World War. While the United Nations (UN) Charter grants every country equal voting rights, there are many other multilateral platforms where the developed countries have a much stronger say over developing countries, such as the IMF and the WB. Both institutions send most of their funds to the developing world, with some arguing that they are as powerful, if not more powerful players in global health than the World Health Organization (WHO) [36], yet there is so little representation from the developing world in leading positions of those two institutions.

Promoting the central role of WHO in global health, safeguarded by the UN Charter agreed upon by all Member States, constitutes a feasible and promising way forward to ensure that institutional powers of major players in global health do not work against the quest for equity. The global health society ought to recognize the WHO as the central player in global health and work on ways to support its independence and solve the many barriers it faces that prevent it from taking on that responsibility [37].

### A pending paradigm shift

One fundamental premise of global health is that many of the developing countries do not have the solution to the health problems they have. And that is a premise that needs to be revisited from the post-colonial and neo-colonial lens.

In her recent post, Dr. Yerramilli argued that the out flow of funds from the poor nations to the rich nations, in terms of repatriation of profits for multinational corporations and etc. outweigh the aid for health sent from rich nations to poor nations every year [38]. The same goes for the human resource flow between rich and poor countries: yes, rich countries sent trained experts to poor countries to provide technical support for the health sector, but every year, a much larger number of top health professionals are leaving poor nations to go to rich nations and form the main workforce of their health care systems, some by solicitous policies that specifically targets health professionals from developing countries [39]. To look at those two issues from another angle, one would see that: developing countries already have all the resources needed to solve their health issues, in both monetary and human capitals. But those solutions were first taken away from them, and then come back in a much smaller and fragmented form with lots of strings attached, and with a new name called global health.

People working in global health have to jump out of the sector silo where they only see the resources from the developed countries be sent to solve the problems of the developing countries. Those activities perpetrate an image of victimhood for the developing world that is false, inappropriate and unjust. Researchers and scholars need to closely study and inspect the true tie: are the developed economies generously giving back to the developing economies, or were they built from the hard work of the developing economies to begin with?

As is shown in the figure, the three levels of colonial remnant in global health form a hierarchy based on how discrete they are. While the colonial remnant in daily practices can be easily noticed by examining the numbers of publication by origin, flow of capital and human resources, representation of leadership positions in international organizations, etc., colonial remnant in organizational structures might not be that obvious. One would need to study the decision-making process, trace down the source of funding, and identify the true constituency to notice that the setup of many organizations operating in global health today follows the same colonial structure. Last but not least, the colonial mindset is deeply embedded in how people as global health workers, advocates, researchers and decision makers think and assume that have subconsciously made us less sensitive to the colonial remnant in daily practices and in the organizational setup.

## Decolonizing global health: under what guidance?

The decolonizing global health movement will not succeed without the decolonization of the world’s political economy. This involves removing the underpinning social-economic inequity that was exacerbated by colonization and has never been undone since. For all scholars and professionals working to push for a more equitable and just world, we say, the end goal is, first and foremost, the equitable economic ownership of the global wealth by all human beings. The day that happens, the day colonialism becomes a thing of the past and the day any form of supremacy will find no room to survive on our Earth.

In recognizing that principle, the research and practice of global health have to go hand in hand with the socioeconomic development of all nations. Health, as one of the fundamental human rights, cannot stand alone without a peaceful and prosperous society. A society where people have to pay for most of their life essential products made by foreign owned manufacturers and sold in foreign owned stores would not have enough tax revenue to cover their own health needs and have to negotiate with donors on what the real health priorities of their country are. A society failing to provide enough well-paid jobs for health professionals will lose their best talents and then have to work with experts paid for by donors to convince them what should be the real health priorities of their country. Economic development has to take place for global health not to be served as temporary blood infusion into a body that has chronic internal hemorrhage.

Fortunately, the world has made a few constructive steps forward from colonial times, and multiple international disputes resolution platforms have been established to prevent and address large scale human atrocities, including the United Nations. Although it was not set up to end colonization in specific, the fundamental principle of “sovereign equality of all its members” in the UN Charter^40^ puts it in a legitimate position to lead the world to complete its decolonization process. We hope, the three levels of colonial remnant we categorized in this article can serve as a conceptual tool and pave ways for more future research in this area, to identify and specify problems, and most importantly, to come up with practical and effective solutions.

Decolonizing global health requires coordinated efforts from multiple sectors in which health becomes a central consideration. When forming an alliance is not feasible, global health programs and projects should at least pave ways or provide insights for the future economic and social development of the communities they serve. The decolonizing global health movement can, and ought to, join all movements that call for equity in other walks of life, especially equity in socioeconomic ownership of our own world. We all need to do the work to make global health truly global.

## Data Availability

Not applicable.

## Abbreviations

HICs: High-income countries
HSDPs: Health Strategic Development Plans
ICSOs: International Civil Society Organizations
IMF: International Monetary Fund
LMICs: Low- and middle-income countries
NCDs: Non-communicable diseases
TB: Tuberculosis
WB: World Bank
WHO: World Health Organization
UN: United Nations
